# Evaluación de la efectividad de la prueba rápida OptiMAL-IT™ para el seguimiento de pacientes con diagnóstico de malaria en la Amazonía peruana

**DOI:** 10.7705/biomedica.6079

**Published:** 2022-03-01

**Authors:** Nancy Arróspide, Hernán Sanabria, William J. Araujo-Banchon

**Affiliations:** 1 Universidad Nacional Mayor de San Marcos, Lima, Perú Universidad Nacional Mayor de San Marcos Universidad Nacional Mayor de San Marcos Lima Peru; 2 Universidad Continental, Lima, Perú Universidad Continental Lima Perú

**Keywords:** Plasmodium, malaria/diagnóstico, cuidados posteriores, efectividad, Perú, Plasmodium, malaria/diagnosis, aftercare, effectiveness, Perú

## Abstract

**Introducción.:**

En Perú, la microscopía óptica con gota gruesa continúa utilizándose en el seguimiento de los pacientes con malaria o paludismo. Esta prueba es sencilla, pero requiere de equipamiento microscópico y personal idóneo que realice la lectura de las muestras. Los estudios sugieren que la prueba rápida OptiMAL-IT™ es una opción para dicho seguimiento.

**Objetivo.:**

Evaluar la efectividad de OptiMAL-IT™ como test de seguimiento en pacientes con malaria en áreas endémicas del Perú.

**Materiales y métodos.:**

Se hizo un estudio observacional, transversal y analítico de pruebas diagnósticas en pacientes con malaria. Se seleccionó a todos los pacientes que cumplían con los criterios de inclusión, procedentes de diferentes establecimientos de salud de los departamentos peruanos de San Martín y Loreto. El diagnóstico se hizo mediante microscopía óptica con gota gruesa y la prueba rápida de diagnóstico inmunocromatográfico OptiMAL-IT™ en los días 2, 3, 7 y 14 para *Plasmodium vivax* y hasta el día 21 de seguimiento para *Plasmodium falciparum*. Se calculó el porcentaje de los correctamente clasificados y los valores predictivos, y se compararon los resultados de la selva occidental y la selva oriental mediante ji al cuadrado o prueba exacta de Fisher.

**Resultados.:**

Se registraron 262 pacientes de San Martín y 302 de Loreto. Los porcentajes correctamente clasificados y el valor predictivo negativo fueron superiores a 92,0 y 93,0 %, respectivamente, a partir del tercer día de seguimiento; no se encontraron diferencias estadísticas en los resultados obtenidos en la Amazonía occidental y los de la oriental.

**Conclusiones.:**

La prueba OptiMAL-IT™ sería efectiva como test de seguimiento en los pacientes con diagnóstico de malaria en áreas endémicas del Perú.

## Abstract (en)

**Introduction:**

In Peru, optical microscopy with the thick smear test continues to be performed for the follow-up of malaria patients. This test is simple but it requires microscopic equipment and suitable staff to perform the reading of the samples. Studies suggest that the rapid OptiMAL-IT™ test is an option for follow-up.

**Objective:**

To evaluate the effectiveness of OptiMAL-IT™ as a follow-up test in malaria patients in endemic areas of Perú.

**Materials and methods:**

We conducted an observational, analytical cross-sectional study of diagnostic tests performed in patients with malaria. We selected all the patients attending different health facilities in the Peruvian departments of San Martín and Loreto who met the inclusion criteria. Optical microscopy with thick smear and OptiMAL-IT™ was used on days 2, 3, 7, and 14 for *Plasmodium vivax* and until day 21 of follow-up for *Plasmodium falciparum*. Percentages of correctly classified samples and predictive values were calculated, and the results were compared between the western jungle and the eastern jungle using Chi2 or Fisher's exact tests.

**Results:**

We registered 262 patients from San Martín and 302 from Loreto. The percentage of correctly classified cases and the negative predictive value were higher than 92.0% and 93,0%, respectively, from the third day of follow-up; no statistical differences were found in the results obtained from the western jungle and those from the eastern jungle.

**Conclusions:**

The OptiMAL-IT™ test would be effective as a follow-up test in patients diagnosed with malaria in endemic areas of Perú.

La malaria o paludismo es una enfermedad transmitida por la picadura de mosquitos hembra del género *Anopheles*, los cuales inoculan en el ser humano el protozoario del género *Plasmodium*^1^. Se trata de una enfermedad potencialmente mortal que, según la Organización Mundial de la Salud (OMS), registró en el 2019 una prevalencia mundial de 230 millones de casos y causó la muerte de 409.000 personas ^2^. En el 2019, el Centro Nacional de Epidemiología, Prevención y Control de Enfermedades del Perú (CDC Perú) reportó 23.869 casos de malaria y 5 muertes por cada 100.000 habitantes ^3^. Estas cifras representan casi la cuarta parte de lo que se reportaba a inicios de la década del 2000, con 81.697 casos de malaria a nivel nacional y 6 defunciones (2004) ^4^.

El método diagnóstico de referencia para la malaria es el examen microscópico de la gota gruesa ^5^. Esta prueba tiene una sensibilidad del 100 % (IC_95%_ 99,23-100), una especificidad del 97,14 % (IC_95%_ 90,10-100) ^6^, y es sencilla y barata (13,88 soles, aproximadamente 3,50 dólares) ^7^. Sin embargo, requiere de equipamiento microscópico y personal capacitado que realice la lectura de las muestras ^8^^,^^9^. Todo esto hace que su implementación en lugares de difícil acceso sea poco rentable ^9^. En Perú, dicha prueba se usa como método diagnóstico con un programa de evaluación externa, tal como aconseja la OMS, el cual es evaluado anualmente por la red nacional de establecimientos liderados por el Instituto Nacional de Salud, adscrito al Ministerio de Salud. La microscopía óptica continúa empleándose para el seguimiento de los pacientes ya diagnosticados ^10^.

En estudios realizados en Perú ^11^^,^^12^ y en Colombia ^6^, se ha reportado que la prueba rápida con tira reactiva (OptiMAL-IT™) es una opción efectiva por su sensibilidad y especificidad en el diagnóstico de malaria, lo que se ha corroborado en el seguimiento de la enfermedad en estudios internacionales, aunque ninguno en el continente americano ^13^^,^^14^.

La prueba OptiMAL-IT™ consiste en una tira reactiva para la prueba inmunocromatográfica ^15^. Utiliza anticuerpos monoclonales para detectar los epítopos de la enzima lactato-deshidrogenasa de cualquiera de las formas (sexuadas o asexuadas) de especies de *Plasmodium* (pLDH) ^15^^,^^16^. En este contexto, el objetivo del presente estudio fue evaluar su efectividad como test para el seguimiento de los pacientes con diagnóstico de malaria en áreas endémicas de Perú.

## Materiales y métodos

### Diseño del estudio y pacientes

Se hizo un estudio observacional, transversal y analítico de pruebas diagnósticas a partir de muestras de sangre tomadas a pacientes atendidos en establecimientos de salud de los departamentos peruanos de San Martín y Loreto. Se incluyeron todos los pacientes febriles mayores de dos años que asistieron entre marzo y diciembre del 2004 a los establecimientos de salud con resultado positivo en la prueba de gota gruesa y a quienes se les había hecho la prueba OptiMAL-IT™; debían haber sido diagnosticados únicamente con infección por P. vivax o por *P. falciparum* y ser residentes de los departamentos de San Martín o Loreto. Se excluyeron aquellos con seguimiento programado incompleto, mujeres gestantes y pacientes con infecciones mixtas o con signos de peligro de malaria grave.

### Procedimientos

La recolección de los datos se coordinó con las direcciones regionales de salud de los departamentos de San Martín y Loreto y, posteriormente, se tuvo acceso a los establecimientos de salud de Barranquita, Bellavista, Pongo Caynarachi, San Miguel de Achinamiza, Alianza, Pampa Hermosa, San Antonio, Padre Cocha y 9 de Octubre. Se seleccionaron todos los pacientes que cumplieron con los criterios de elegibilidad.

Después de identificar a un paciente febril, el establecimiento de salud procedía a hacer el diagnóstico parasitológico con el método de gota gruesa y microscopía según los protocolos del Ministerio de Salud ^17^.

Los pacientes que dieron positivos para malaria recibieron su tratamiento como parte de la Estrategia Sanitaria Nacional de Prevención y Control de las Enfermedades Metaxénicas: los portadores de *P. falciparum* recibieron mefloquina y artesunato, y los portadores de *P. vivax*, primaquina y cloroquina.

Ya seleccionados para el estudio, se les tomaron dos muestras de sangre por punción digital según las recomendaciones del Ministerio de Salud ^17^. Una muestra se utilizó para el cálculo de la densidad parasitaria (parásitos/ μl) y, la otra, para la evaluación de la pLDH mediante OptiMAL-IT™. La tira reactiva de esta prueba presenta tres bandas que aparecen cuando se detecta el antígeno pLDH: el anticuerpo caprino antirratón sirve como banda de control (C); la banda anti-pLDH inespecífica reacciona contra *P. falciparum, P. vivax, P. malariae* o *P. ovale* (P), y la banda anti-pLDH específica contra *P. falciparum* (Pf) ^15^. Si se colorea C, la prueba es negativa; si se colorean C y P, la reacción es positiva para *Plasmodium* no *falciparum*; si se colorean C, P y Pf, la reacción es positiva para *P. falciparum*^15^^,^^18^ (figura 1). Ambas pruebas estuvieron a cargo de personal de laboratorio calificado y se ajustaron a las directrices de control de calidad para el diagnóstico de malaria recomendados por la OMS ^19^. Este mismo proceso se hizo en los días 2, 3, 7 y 14 para el seguimiento de los pacientes con diagnóstico de malaria por *P. vivax*. En el caso de los aquellos con diagnóstico de malaria por *P. falciparum*, se hizo una toma adicional el día 21 ^17^^,^^20^.

Las muestras fueron remitidas al Instituto Nacional de Salud para corroborar, en ciego, los resultados de la microscopía con gota gruesa y la segunda lectura de la densidad parasitaria. Si la densidad parasitaria calculada por el personal del Instituto tenía una diferencia menor del 50 % de la calculada por el personal del establecimiento de salud de la selva, se procedía a obtener un promedio de ambas densidades. Si la diferencia era mayor del 50 %, se remitía a un tercer evaluador (también cegado) para el cálculo de una nueva densidad parasitaria ^20^^,^^21^.

Se diseñó una ficha de recolección de los datos de las primeras lecturas realizadas en los establecimientos de salud seleccionados, así como de las segundas lecturas del laboratorio de referencia nacional del Instituto Nacional de Salud, lo cual figura todo en sus registros. Posteriormente, se elaboró una base de datos con Microsoft Excel 2010.

### Variables

El resultado de la gota gruesa se definió como la visualización microscópica del parásito en cualquiera de sus etapas (sexuadas o asexuadas), lo que permitió discriminar entre *P. falciparum* y *P. vivax*. El resultado de la prueba OptiMAL-IT™ se estableció según el número de bandas en la tira reactiva, lo que permitió diferenciar entre malaria por *P. falciparum* (banda C + P + Pf) y malaria por *P. vivax* (banda C + P) ^18^.

Los días de seguimiento se definieron como aquellos en que se hacía el control del paciente con exámenes de laboratorio por gota gruesa y OptiMAL- IT™: días 2, 3, 7, 14 y 21. El número de parásitos en fase asexual se definió como el número de trofozoítos visibles durante la evaluación microscópica de la gota gruesa. El número de leucocitos en las formas asexuadas se definió como aquel que debía observarse para establecer el número de parásitos en fase asexual encontrado en el recuento parasitario. El número de parásitos en fase sexual se definió como el número de gametocitos visibles durante la evaluación microscópica de la gota gruesa. El número de leucocitos en los parásitos en fase sexual se definió como el número de leucocitos que debía observarse para establecer el número de parásitos en esta fase encontrado en el recuento parasitario.

La densidad parasitaria (parásitos/μl) se estimó considerando el valor de referencia de 6.000 leucocitos, es decir, se multiplicó el número de parásitos por 6.000 y el resultado se dividió entre el número de leucocitos ^17^. Para el caso de las formas asexuadas, las unidades se expresaron en trofozoítos/μl de sangre, en tanto que, en el caso de las formas sexuadas, se expresaron en gametocitos/μl de sangre.

### Análisis estadístico descriptivo

Las variables categóricas se expresaron en frecuencias absolutas y relativas y fueron: el día de tratamiento, el resultado de la gota gruesa y el de la prueba OptiMAL-IT™. Las variables numéricas incluyeron la densidad parasitaria de las formas asexuadas y de las sexuadas, y se expresaron en rangos.

Para evaluar el desempeño de la prueba OptiMAL- IT™, se calculó su porcentaje de aciertos y se lo comparó con la gota gruesa. Este desempeño se denominó porcentaje de correctamente clasificados. Los cálculos se hicieron de forma global según cada especie de *Plasmodium* y según el área amazónica (selva occidental y selva oriental); estos cálculos también se hicieron para los días de tratamiento 2, 3, 7, 14 y 21.

### Análisis estadístico analítico

Se compararon los porcentajes de correctamente clasificados de la selva occidental y los de la selva oriental mediante las pruebas de ji al cuadrado o exacta de Fisher dependiendo de los resultados de las frecuencias esperadas. Se consideró un alfa de 0,05 y un intervalo de confianza (IC) del 95 %. Como soporte informático se utilizaron los programas Microsoft Excel 2010 y Stata™, versión 12.1.

### Consideraciones éticas

El estudio fue aprobado por el Comité de Ética del INS (código C-12-2002/ INS). Se garantizó la confidencialidad de los datos recolectados, los cuales se codificaron para impedir el futuro reconocimiento de los pacientes por parte de los investigadores, así como la autonomía de los participantes mediante la firma de un consentimiento informado.

## Resultados

### Características de los establecimientos de salud

En el departamento de San Martín los establecimientos de salud de los cuales provenían los datos de los pacientes con diagnóstico de malaria fueron Barranquita (n=8), Bellavista (n=98), Pongo Caynarachi (n=33), San Miguel de Achinamiza (n=46) y Alianza (n=77). En Loreto, fueron Pampa Hermosa (n=113), San Antonio (n=85), Padre Cocha (n=82) y 9 de Octubre (n=22). Se consideró que los establecimientos de salud del departamento de San Martín y un establecimiento ubicado en el departamento de Loreto (Pampa Hermosa), correspondían a la selva occidental, en tanto que el resto se consideró parte de la selva oriental.

Se registraron 564 pacientes con diagnóstico de malaria (262 de San Martín y 302 de Loreto, 375 de la selva occidental y 189 de la oriental), a quienes se les hicieron las dos pruebas de laboratorio. Si bien fueron 81 los pacientes con diagnóstico inicial de *P. falciparum*, solo en 76 fue posible hacer el control en el día 21.

### Evaluación del seguimiento con las dos pruebas diagnósticas (gota gruesa y OptiMAL-IT™)

En los cuadros 1 a 4 se presentan los porcentajes de correctamente clasificados y sus valores predictivos según el día de seguimiento de los pacientes con malaria. Se evidenció que más del 97 % de las personas de la Amazonía peruana con diagnóstico inicial de malaria por *P. falciparum*, registraron los mismos resultados con las dos pruebas diagnósticas en todos los días de seguimiento. Asimismo, más del 98 % de los participantes con resultado negativo para *P. falciparum* en la gota gruesa también fueron negativos en la OptimMAL-IT™. Un comportamiento similar en cuanto al correctamente clasificados y el valor predictivo negativo (VPN) se observó en los subgrupos de la Amazonía occidental y la oriental. No se encontraron diferencias significativas en los porcentajes de correctamente clasificados ni en los VPN entre los subgrupos de pacientes de la Amazonía occidental y la oriental.

**Cuadro 1:** Día 2 de seguimiento de los pacientes con diagnóstico de malaria

Prueba OptiMAL-IT™	Gota gruesa		VPP (%)	VPN (%)	CC (%)
	Positivo	Negativo			
Amazonia peruana (n=564)					
Global*					
Positivo	17	17	50,00	87,74	85,46
Negativo	65	465			
*P. vivax*					
Positivo	12	13	48,00	89,42	87,59
Negativo	57	482			
*P. falciparum*					
Positivo	5	4	55.56	98,56	97,87
Negativo	8	547			
Amazonia peruana occidental (n=375)					
Global*					
Positivo	7	5	58,33	87,05	86,13**
Negativo	47	316			colspan="2">
*P. vivax*					
Positivo	5	3	62,50	88.83	88,27***
Negativo	41	326			
*P. falciparum*					
Positivo	2	2	50,00	98,38	97,87****
Negativo	6	365			
Amazonia peruana oriental (n=189)					
Global*					
Positivo	10	12	45,45	89,22	84,13**
Negativo	18	149			
P. vivax					
Positivo	7	10	41,18	90,70	86,24***
Negativo	16	156			
*P. falciparum*					
Positivo	3	2	60,00	98,91	97,88****
Negativo	2	182				

VPP: valor predictivo positivo; VPN: valor predictivo negativo; CC: correctamente clasificado; selva occidental: Barranquita, Bellavista, Pongo Caynarachi, San Miguel de Achinamiza, Alianza y Pampa Hermosa; selva oriental: San Antonio, Padre Cocha y 9 de Octubre

* Segundo control de malaria independientemente de la especie

** p=0,523 (prueba de ji al cuadrado)

*** p=0,492 (prueba de ji al cuadrado)

**** p>0,05 (prueba exacta de Fisher)

En el tercer día de seguimiento (cuadro 2), más del 93 % de los participantes registraron resultados iguales para todas las especies de *Plasmodium* (con una tendencia mayor para *P. falciparum*), tanto a nivel global como por subgrupos. No se observaron diferencias significativas en los porcentajes de los correctamente clasificados ni en los VPN entre los subgrupos de los pacientes de la Amazonía occidental y oriental.

**Cuadro 2:** Día 3 de seguimiento de los pacientes con diagnóstico de malaria

Prueba OptiMAL-IT™	Gota gruesa		VPP (%)	VPN (%)	CC (%)
	Positivo	Negativo			
Amazonia peruana (n=564)					
Global*					
Positivo	4	8	33,33	94,38	93,09
Negativo	31	521			
*P. vivax*					
Positivo	0	2	0,00	95,55	95,21
Negativo	25	537			
*P. falciparum*					
Positivo	4	6	40,00	98,92	97,87
Negativo	6	548			
Amazonia peruana occidental (n=375)					
Global*					
Positivo	2	2	50,00	93,26	92,80**
Negativo	25	346			
*P. vivax*					
Positivo	0	0	0,00	94,67	94,67***
Negativo	20	355			
*P. falciparum*					
Positivo	2	2	50,00	98,65	98,13****
Negativo	5	366			
Amazonia peruana oriental (n=189)					
Global*					
Positivo	2	6	25,00	96,69	93,65**
Negativo	6	175			
*P. vivax*					
Positivo	0	2	0,00	97,33	96,30***
Negativo	5	182			
*P. falciparum*					
Positivo	2	4	33,33	99,45	97,35****
Negativo	1	182			

VPP: valor predictivo positivo; VPN: valor predictivo negativo; CC: correctamente clasificado; selva occidental: Barranquita, Bellavista, Pongo Caynarachi, San Miguel de Achinamiza, Alianza y Pampa Hermosa; selva oriental: San Antonio, Padre Cocha y 9 de Octubre

* Tercer control de malaria independientemente de la especie

** p=0,707 (prueba de ji al cuadrado)

*** p=0,392 (prueba de ji al cuadrado)

**** p=0,548 (prueba exacta de Fisher)

En los días 7 y 14 de seguimiento (cuadros 3 y 4), más del 95 % de los pacientes registró resultados iguales para todas las especies de *Plasmodium* (con una tendencia superior para *P. falciparum*), tanto en la Amazonía globalmente como por subgrupos. No se observaron diferencias significativas en los porcentajes de correctamente clasificados ni en los VPN entre los subgrupos de la Amazonía occidental y la oriental.

**Cuadro 3:** Día 7 de seguimiento de los pacientes con diagnóstico de malaria

Prueba OptiMAL-IT™	Gota gruesa		VPP (%)	VPN (%)	CC (%)
	Positivo	Negativo			
Amazonía peruana (n=564)					
Global*					
Positivo	4	3	57,14	96,05	95,57
Negativo	22	535			
*P. vivax*					
Positivo	0	1	0,00	96,80	96,63
Negativo	18	545			
*P. falciparum*					
Positivo	4	2	66,67	99,28	98,94
Negativo	4	554			
Amazonía peruana occidental (n=375)					
Global*					
Positivo	1	0	100,00	95,19	95,20**
Negativo	18	356			
*P. vivax*					
Positivo	0	0	0,00	96,00	96,00***
Negativo	15	360			
*P. falciparum*					
Positivo	1	0	100,00	99,20	99,20****
Negativo	3	372			
Amazonía peruana oriental (n=189)					
Global*					
Positivo	3	3	50,00	97,81	96,30**
Negativo	4	179			
*P. vivax*					
Positivo	0	1	0,00	71,81	97,88***
Negativo	3	185			
*P. falciparum*					
Positivo	3	2	60,0	99,46	98,41****
Negativo	1	183			

VPP: valor predictivo positivo; VPN: valor predictivo negativo; CC: correctamente clasificado; selva occidental: Barranquita, Bellavista, Pongo Caynarachi, San Miguel de Achinamiza, Alianza y Pampa Hermosa; selva oriental: San Antonio, Padre Cocha y 9 de Octubre

* Séptimo control de malaria independientemente de la especie

** p=0,555 (prueba de ji al cuadrado)

*** p=0,244 (prueba de ji al cuadrado)

**** p=0,407 (prueba exacta de Fisher)

**Cuadro 4:** Día 14 de seguimiento de los pacientes con diagnóstico de malaria

Prueba OptiMAL-IT™	Gota gruesa		VPP (%)	VPN (%)	CC (%)
	Positivo	Negativo			
Amazonía peruana (n=564)					
Global*					
Positivo	3	4	42,86	95,59	95,92
Negativo	19	538			
*P. vivax*					
Positivo	1	2	33,33	96,97	96,63
Negativo	17	544			
*P. falciparum*					
Positivo	2	2	50,00	99,64	99,29
Negativo	2	558			
Amazonía peruana occidental (n=375)					
Global*					
Positivo	1	0	100,00	96,26	96,27**
Negativo	14	360			
*P. vivax*					
Positivo	0	0	0,00	96,80	96,80***
Negativo	12	363			
*P. falciparum*					
Positivo	1	0	100,00	99,47	99,47****
Negativo	2	372			
Amazonía peruana oriental (n=189)					
Global*					
Positivo	2	4	33,33	97,27	95,24**
Negativo	5	178			
*P. vivax*					
Positivo	1	2	33,33	97,31	96,30***
Negativo	5	181			
*P. falciparum*					
Positivo	1	2	33,33	100,00	98,94****
Negativo	0	186			

VPP: valor predictivo positivo; VPN: valor predictivo negativo; CC: correctamente clasificado; selva occidental: Barranquita, Bellavista, Pongo Caynarachi, San Miguel de Achinamiza, Alianza y Pampa Hermosa; selva oriental: San Antonio, Padre Cocha y 9 de Octubre

* Día 14 de control de malaria independientemente de la especie

** p=0,560 (prueba de ji al cuadrado)

*** p=0,754 (prueba de ji al cuadrado)

**** p=0,605 (prueba exacta de Fisher)

En cuanto al día 21 de control de los pacientes con *P. falciparum*, 63 muestras correspondieron a los de la selva occidental y 13 a los de la selva oriental. Todas dieron resultados negativos en el examen de gota gruesa y en el OptiMAL-IT™.

### Evaluación de la densidad parasitaria

Las muestras de sangre de los participantes presentaron una densidad parasitaria superior a 100 parásitos/μl en el 98,87 % (n=552) de los casos. En el cuadro 5, se observa la densidad parasitaria máxima contabilizada en cada día de seguimiento. Sin embargo, debe anotarse que la densidad parasitaria de los parásitos sexuados (gametocitos) fue nula en más del 96 % y en cada uno de los días de seguimiento: 96,99 % (n=547) en el día 2; 97,87 % (n=552) en el día 3; 99,11 % (n=559) en el día 7; 99,11 % (n=559) en el día 14, y 100 % (n=76) el día 21. En el caso de las formas asexuadas (trofozoítos), la densidad parasitaria fue cero en más del 85 % y en cada uno de los días de seguimiento: 85,99 % (n=485) en el día 2; 95,74 % (n=540) en el día 3; 96,81 % (n=546) en el día 7; 97,87 % (n=552) en el día 14, y 100 % (n=76) en el día 21.

**Cuadro 5:** Densidad parasitaria máxima según día de seguimiento

Día de examen de seguimiento	Formas asexuadas (trofozoítos/μl)	Formas sexuadas (gametocitos/μl)
2	18.303	1.403
3	647	1.024
7	4.946	384
14	350	23
21	0	0

## Discusión

El sistema de salud peruano sigue utilizando la microscopía óptica como prueba de referencia para el seguimiento de los pacientes con diagnóstico de malaria; sin embargo, se sabe que este método tiene varias limitaciones, entre ellas, la necesidad de contar con personal entrenado en la lectura microscópica de las muestras, el tiempo requerido para hacer la lectura microscópica y la buena preparación de las muestras para asegurar resultados de calidad ^8^.

OptiMAL-IT™ es una prueba de diagnóstico rápida cuyo costo en el mercado peruano es superior al de la microscopía con gota gruesa (aproximadamente 6,80 Vs. 3,50 dólares) ^7^. Sin embargo, sería una prueba más rentable como método de seguimiento alternativo frente a la microscopía, ya que no se requiere un microscopio, no es necesario contar con personal altamente entrenado para su lectura y los resultados se obtienen en 20 minutos ^22^. Por todo ello, sería de mucha utilidad en lugares endémicos para malaria que tienen escasos recursos ^23^.

Existe otro grupo de pruebas de diagnóstico rápido que detecta proteínas ricas en histidina (HRP-2), pero estas arrojan muchos falsos positivos y falsos negativos. Los falsos positivos se explican en estudios que evidencian la presencia del antígeno HRP-2 incluso meses después de la eliminación del parásito ^16^^,^^24^. En una revisión sistemática de 31 publicaciones en el 2018, se evidenció que el 50 % de las pruebas de diagnóstico rápido contra el antígeno HRP-2 seguían siendo positivas 15 días después del tratamiento ^24^.

Las tasas elevadas de positivos en pacientes con tripanosomiasis africana humana o en enfermedades concomitantes con factor reumatoideo, también resultarían en falsos positivos ^25^^,^^26^. Los falsos negativos pueden ocurrir porque la producción de HRP-2 es exclusiva de *P. falciparum*^27^, lo que llevaría a resultados negativos en casos de malaria por *P. vivax*.

En Perú, también circula la especie *P. vivax*^28^ y es más prevalente que *P. falciparum* (relación 4/1 en el 2015) ^29^, por lo que no sería adecuado el uso de las pruebas de diagnóstico rápido que solo detectan las HRP-2. Los falsos negativos también pueden ser consecuencia de la variabilidad de la expresión del gen que codifica la proteína HRP-2 de los parásitos ^30^^-^^32^. En la Amazonía peruana, se han evidenciado deleciones en los genes que codifican esta proteína, lo que conlleva elevadas tasas de falsos negativos y hace inviable el uso de este tipo de PDR en esta área ^33^^,^^34^.

El porcentaje de correctamente clasificados, que traduce el desempeño de la prueba evaluada, fue superior al 90,0 % a partir del tercer día de seguimiento, similar a lo reportado en otros estudios ^14^. Asimismo, el VPN fue superior al 90,0 % en todos los días de seguimiento, lo que permite sugerir que la prueba OptiMAL-IT™ sería una opción que brinda resultados seguros en el seguimiento de pacientes con diagnóstico de malaria, principalmente por *P. falciparum*, con porcentajes de correctamente clasificados superiores al 90 % desde el segundo día de seguimiento y porcentajes de VPN de casi el 100 %.

En el estudio de Griffing, *et al*., la geografía de la selva peruana se dividió en un sector occidental y uno oriental a partir de la distribución de clones específicos de *P. falciparum* en cada uno de ellos ^36^. Esta división se tomó en consideración para las comparaciones geográficas de los resultados en el presente estudio. Los establecimientos de salud pertenecientes al departamento de San Martín (Barranquita, Bellavista, Pongo Caynarachi, San Miguel de Achinamiza y Alianza) se consideraron parte de la selva occidental y se agregó un establecimiento de salud del departamento de Loreto (Pampa Hermosa) debido a su ubicación geográfica cercana a los establecimientos de la región occidental. El resto de los establecimientos de salud de Loreto (San Antonio, Padre Cocha y 9 de Octubre) se consideraron como parte de la selva oriental peruana. No se observaron diferencias significativas en los porcentajes de correctamente clasificados en ninguno de los días de seguimiento ni entre los dos sectores de la selva, lo que permite sugerir la utilidad de la prueba OptiMAL-IT™ indistintamente de la ubicación amazónica.

Tal como lo recomienda la OMS, el uso de la prueba OptiMAL-IT™ para el seguimiento no excluye el control de calidad mediante microscopía óptica de la gota gruesa. En este sentido, los establecimientos de salud de los pacientes con malaria deberán enviar las muestras de sangre a la institución de referencia ^19^ para así garantizar la correcta implementación de las PDR en los programas de control de malaria. Asimismo, deberán contar con profesionales entrenados en el uso de los kits de PDR, los cuales deberán de conservarse apropiadamente para evitar el deterioro de los anticuerpos monoclonales.

Con base en estos hallazgos, se recomienda que en zonas endémicas de la Amazonía peruana se utilicen las pruebas de diagnóstico rápido que detectan el pLDH, entre ellas, la OptiMAL-IT™, como método de seguimiento de los casos de pacientes con malaria, pues arrojan correctamente clasificados y VPN adecuados, y permitirían suplir de forma más eficiente la función de las pruebas con gota gruesa. Estas pruebas de diagnóstico rápido pueden usarse cuando no se tenga acceso a microscopía de la gota gruesa o cuando se carezca de microscopistas entrenados.

Debe precisarse que el uso de pruebas secuenciales (en serie) con microscopía óptica para el diagnóstico de los pacientes con malaria participantes en estudios, ha generado pérdida de sensibilidad neta y ganancia de especificidad neta. Por otro lado, el uso de pruebas simultáneas en cada día de seguimiento aumentó la sensibilidad neta y disminuyó la especificidad neta ^37^.

En el presente estudio, no se encontraron limitaciones debidas a la presencia de pruebas secuenciales, puesto que todos los pacientes positivos en la gota gruesa en su respectivo establecimiento de salud también lo fueron en la evaluación de su parasitemia en el día de ingreso al estudio (día cero). Es probable que la especificidad neta sí haya disminuido dado que para el seguimiento se emplearon las pruebas de gota gruesa y OptiMAL- IT™ conjuntamente. Sin embargo, esto no se consideró relevante porque el objetivo del estudio no fue cuantificar la validez del diagnóstico (sensibilidad y especificidad) ^37^ sino, más bien, cuantificar la confiabilidad ^37^ de la prueba OptiMAL-IT™ al brindar resultados consistentes con el estado de salud de los pacientes (valores predictivos).

Otras limitaciones que deben considerarse es que debieron excluirse del estudio los pacientes que no asistieron al seguimiento en los días 14 o 21, ya fuera porque dejaron de acudir o porque no fue posible ubicarlos en la jurisdicción del establecimiento de salud. Por ello, no es posible extrapolar los resultados a quienes no cumplieron con el tratamiento propuesto. El estudio excluyó a las mujeres gestantes, por lo que se requieren estudios enfocados en este grupo poblacional, pero sí incluyó adultos y niños, de manera que las decisiones específicas para la población pediátrica deben tomarse con cautela. Las comparaciones entre la selva occidental y la oriental son el resultado de un análisis de subgrupos, por lo que se sugieren nuevos estudios en los que los cálculos de tamaño de la muestra se ajusten a la hipótesis de la existencia de diferencias entre estas ubicaciones geográficas de la selva peruana.
